# Clot Structure and Fibrinolysis in Thrombosis and Hemostasis

**DOI:** 10.1155/2017/4645137

**Published:** 2017-11-15

**Authors:** Zsuzsa Bagoly, Robert A. S. Ariëns, Dingeman C. Rijken, Marlien Pieters, Alisa S. Wolberg

**Affiliations:** ^1^Department of Laboratory Medicine, Division of Clinical Laboratory Sciences and MTA-DE Cerebrovascular and Neurodegenerative Research Group, University of Debrecen, Debrecen, Hungary; ^2^Leeds Institute of Cardiovascular and Metabolic Medicine, University of Leeds, Leeds, UK; ^3^Department of Hematology, Erasmus University Medical Center, Rotterdam, Netherlands; ^4^Centre of Excellence for Nutrition (CEN), North-West University, Potchefstroom, South Africa; ^5^Department of Pathology and Laboratory Medicine and McAllister Heart Institute, University of North Carolina, Chapel Hill, NC, USA

*“Inimicum quamvis humilem metuendum est”* (“An enemy, however small, is to be feared”).

Today, thrombotic disorders are major contributors to the global burden of disease [1]. The formation of a thrombus, however small, is the common pathology underlying devastating illnesses including ischemic heart disease, ischemic stroke, and venous thromboembolism. Due to the high rates of mortality and morbidity associated with arterial and venous thromboembolism, great efforts have been made in the past years to expand our knowledge on the biological, physical, and chemical features of the blood clot. In this special issue, original research articles and reviews focus on most recent advances on fibrin clot characteristics, mechanisms of clot formation/dissolution, and related clinical conditions.

Fibrin fibers constitute one of the major structural components of the blood clot, providing the clot with a three-dimensional polymeric protein network that imparts considerable elastic strength, with the mechanical and structural properties thereof influencing morbidity and mortality rates associated with thrombotic events. Surprisingly, the process of lateral association of protofibrils into fibrin fibers is still poorly understood. In the research paper by W. Li et al., the authors aim to gain insights into the relatively less studied internal structure of fibrin fibers and describe a novel fiber model. Based on experiments using fluorescence intensity and force (modulus) measurements, they propose that protofibrils are densely packed at the fiber center, but are sparse and loosely connected towards the fiber periphery. The clinical implications of abnormal fibrin clot properties are described in a review article by A. Undas. It has been known for some time that fibrin clots that are composed of thinner, highly branched fibers are less permeable, more rigid, and less susceptible to dissolution by fibrinolytic proteins. A. Undas provides a timely update on the so-called prothrombotic fibrin clot phenotype and its relevance to venous thromboembolism. Listing experimental and clinical studies, the author describes a number of clot structure modifiers implicated in the occurrence of the prothrombotic fibrin clot phenotype and the resulting clot characteristics accounting for a potential risk for thromboembolism.

Not only the formation, but also the dissolution of the fibrin clot is dependent on biochemical and biophysical processes. The review article by N. E. Hudson highlights the importance of understanding fibrinolysis in the light of fibrin biophysical characteristics. The review considers the impact of several structural and mechanical parameters on lytic rates. The author summarizes the newest findings with the expectation that improved understanding of the connection between the biophysical aspects of fibrin and fibrinolytic rates could lead to novel strategies in the development of future fibrinolytic therapies. As an example, the original research by A. Tanka-Salamon et al. describes a novel thrombolytic approach. The purpose of the work was to prepare a phospholipid-based thermosensitive nanocarrier, in which trypsin is attached to the inner leaflet of the bilayer shell of the liposome, and to characterize this new tool in terms of structure and proteolytic efficiency. The authors show that the lytic efficiency of trypsin depends on heat-dependent release from thermosensitive liposomes. The fibrinolytic efficiency of these liposomes was found to be improved in the dynamic fibrinolytic assay under conditions of permeation-driven fibrinolysis. Because intravascular thrombi are exposed to permeation forces, these properties of the construct suggest that it could be a successful candidate as a therapeutic tool, the utility of which deserves further investigation.

A key player in the inhibition of fibrinolysis is activated factor XIII (FXIIIa). An interesting review by D. C. Rijken and S. Uitte de Willige focuses on the impact of this transglutaminase on fibrinolysis. The inhibitory effects of FXIIIa-mediated cross-links on fibrinolysis are summarized and differences in the crosslinking of purified fibrin, plasma, or whole blood clots are highlighted. The potential effect of clot compaction and clot retraction on the inhibition of lysis by FXIIIa and the pathophysiologic aspects of FXIIIa-mediated cross-links are also explained. The catalytic A subunit of FXIII (FXIII-A) is present not only in plasma but also intracellularly in several human cells, suggesting effects of this protein are not restricted to hemostasis. Understanding the roles of FXIII-A outside of the coagulation system is an intriguing area of research with implications for many clinical conditions and pathophysiologic processes including wound healing, angiogenesis, atherosclerosis, and malignancies. In their review paper, L. Paragh and D. Törőcsik summarize current knowledge on intracellular FXIII-A in wound healing, angiogenesis, and various dermatopathologic conditions.

The pathophysiology of increased thrombotic risk in various clinical conditions was studied in two original papers in this issue. N. K. Tóth et al. examined local, intracardiac hemostasis and fibrinolysis abnormalities that are associated with atrial fibrillation (AF) and increase the risk of thromboembolism. After measuring a comprehensive set of fibrinolytic and hemostasis proteins in intracardiac and peripheral blood samples from AF patients and non-AF controls, the authors concluded that AF patients have elevated factor VIII and von Willebrand factor levels, present in the intracardiac and peripheral environment as well, possibly contributing to the increased thrombotic risk associated with this disorder. S. C. Meyer et al. investigated whether anti-platelet factor 4 (PF4)/heparin antibodies contribute to the increased thrombotic risk observed in myeloproliferative neoplasms. They conclude that thrombotic risk increases in anti-PF4/heparin IgG-positive polycythemia vera, reflecting potential clinical implications and calling for larger, confirmatory cohorts.

Clot formation* in vivo* occurs in a complex environment. Whole blood clots contain cells and cell-derived components that greatly influence thrombus size, composition, and stability. In their timely and comprehensive review, J. Kappelmayer and B. Nagy Jr. summarize central function of selectins and their ligands and their roles as key mediators in cellular events during the development of thrombotic and malignant conditions. The role of platelets in clot formation, including thrombin generation and clot contraction/retraction, is another major focus in hemostasis and thrombosis research. R. Hudák et al. describe the effect of the phosphatase inhibitor calyculin-A on various platelet activation mechanisms contributing to clot formation, clot retraction, and thrombin generation and conclude that this inhibitor could serve as a useful tool in experimental studies.

We are confident that papers in this special issue will be of interest and relevance to those involved in experimental and clinical fields related to thrombosis and hemostasis. We hope that the published articles will provide ideas and inspiration to those dedicated to understand the pathophysiologic mechanisms of clot formation and lysis, and will inspire new ideas for diagnosing, treating, and ultimately preventing thrombotic disorders in the future.
